# ETV in infancy and childhood below 2 years of age for treatment of hydrocephalus

**DOI:** 10.1007/s00381-020-04585-8

**Published:** 2020-03-28

**Authors:** Ahmed El Damaty, Sascha Marx, Gesa Cohrs, Marcus Vollmer, Ahmed Eltanahy, Ehab El Refaee, Joerg Baldauf, Steffen Fleck, Heidi Baechli, Ahmed Zohdi, Michael Synowitz, Andreas Unterberg, Henry W. S. Schroeder

**Affiliations:** 1 Department of Neurosurgery, Heidelberg University Hospital, Im Neuenheimer Feld 400, 69120 Heidelberg, Germany; 2 Department of Neurosurgery, University Medicine Greifswald, Greifswald, Germany; 3 Department of Neurosurgery, University Medical Center Schleswig-Holstein (UKSH), Campus Kiel, Kiel, Germany; 4 Institute of Bioinformatics, University Medicine Greifswald, Greifswald, Germany; 5 Mansoura University School of Medicine, Mansoura, Egypt; 6 Department of Experimental Medical Sciences, Faculty of Medicine, Lund University, Lund, Sweden; 7 Department of Neurosurgery, Cairo University, Cairo, Egypt

**Keywords:** Aqueduct stenosis, Endoscopic third ventriculostomy, Obstructive hydrocephalus, Post-hemorrhagic

## Abstract

**Purpose:**

Age and etiology play a crucial role in success of endoscopic third ventriculostomy (ETV) as a treatment of obstructive hydrocephalus. Outcome is worse in infants, and controversies still exist whether ETV is superior to shunt placement. We retrospectively analyzed 70 patients below 2 years from 4 different centers treated with ETV and assessed success.

**Methods:**

Children < 2 years who received an ETV within 1994–2018 were included. Patients were classified according to age and etiology; < 3, 4–12, and 13–24 months, etiologically; aqueductal stenosis, post-hemorrhagic-hydrocephalus (PHH), tumor-related, fourth ventricle outflow obstruction, with Chiari-type II and following CSF infection. We investigated statistically the predictors for ETV success through computing Kaplan-Meier estimates using patient’s follow-up time and time to ETV failure.

**Results:**

We collected 70 patients. ETV success rate was 41.4%. The highest rate was in tumor-related hydrocephalus and fourth ventricle outlet obstruction (62.5%, 60%) and the lowest rate was in Chiari-type II and following infection (16.7%, 0%). The below 3 months age group showed relatively lower success rate (33.3%) in comparison to older groups which showed similar results (46.4%, 46.6%). Statistically, a previous VP shunt was a predictor for failure (*p* value < 0.05).

**Conclusion:**

Factors suggesting a high possibility of failure were age < 3 months and etiology such as Chiari-type II or following infection. Altered CSF dynamics in patients with PHH and under-developed arachnoid villi may play a role in ETV failure. We do not recommend ETV as first line in children < 3 months of age or in case of Chiari II or following infection.

## Introduction

The optimal treatment for hydrocephalus in infants is still not definitively determined [1–7]. Despite the advances that have been achieved recently in the field of neuroendoscopy and shunt hardware, the treatment of hydrocephalus in infants remains one of the most difficult and challenging situations faced by neurosurgeons. Shunts have been used for long to divert CSF in patients with hydrocephalus whether obstructive or communicating. Nowadays, many patients with hydrocephalus are considered candidates for endoscopic third ventriculostomy (ETV) that has gained acceptance in the last 20 years [8].

According to previous reports, age, etiology, and experience of the surgeon are important factors that determine success and complication rates of ETV [9]. Higher success rates, reaching 90%, in some studies have been reported in infants with aqueductal stenosis [10–16]. Lower success rates were reported in patients with postinfectious and post-hemorrhagic hydrocephalus (PHH), and also with prior ventriculoperitoneal (VP) shunt failures [13, 14, 17–20]. Infants have a lower success chance according to Kulkarni et al. [21] suggested score of ETV success considering the age factor. In our study, we report on the success rate of ETV in infants below 2 years of age through our combined experience from 4 university centers.

## Patients and methods

The study was approved by all local ethic boards. According to this approval, patient consent was not necessary because of the retrospective nature of the study. All patients that have received an endoscopic third ventriculocisternostomy and were below 2 years of age at the time of surgery regardless the etiology within the time period from December 1994 to December 2018 were identified out of prospectively collected databases. A thorough review of all medical records has been done consecutively. ETV was either a primary treatment or during the course of disease as an alternative to VP shunts. Cases were collected from 4 University Hospitals as a collaborative experience in obstructive hydrocephalus in infants. Follow-up was documented after clinical examination in outpatient clinics. ETV was considered successful whenever the patient clinically improved or was at least clinically stable without the need for a Re-ETV or a VP shunt. In case of failure of ETV, according to surgeon preference a Re-ETV was considered in some cases before taking the decision of applying a VP shunt.

In order to analyze the success rate, we classified the patients according to age and etiology. According to age, we divided the patients into 3 groups: group I with age 0–3 months, group II with age 4–12 months, and group III with age of 13–24 months at the time of ETV. According to etiology, we divided the patients into 6 groups: idiopathic aqueductal stenosis; PHH; tumor-related hydrocephalus (whether in brainstem or fourth ventricle); fourth ventricle outflow obstruction, i.e., tetraventricular hydrocephalus, hydrocephalus associated with Chiari malformation-type II, and hydrocephalus following CSF infection.

In the statistical analysis, predictors for the ETV success have been investigated. Therefore, we used the patient’s follow-up time and the time to the ETV failure for computing Kaplan-Meier estimates with censored data. Log-rank tests were conducted to check the hypothesis of the equality of event time distributions. These results are printed along with the Kaplan-Maier estimator. Because of non-proportional hazards and since the majority of events happened within 6 months after the ETV surgery, univariable and multivariable analysis is based on logistic regression with the 6 month’s success as a binary response. We computed odds ratios with 95% confidence intervals (95% CI) and age was included either as a continuous or as a categorical variable. Moreover, the cohort was divided into patients with and without 6 months success and the number of patients matching gender, diagnosis, and previous shunt, and age group are presented in a cross table. We compared the numbers using the exact Fisher test or Chi-squared test as appropriate and *p* values are shown in a separate column.

## Results

### Patient characteristics

In the present study, 73 ETVs have been done in 70 patients (35 girls, 35 boys, mean age 6.3 months, range from 14 days to 24 months). Three patients received a Re-do ETV. Twenty-seven patients were below 3 months age at time of procedure (38.6%), 28 were 4–12 months age (40%), and 15 were 13–24 months age (21.4%). Etiologically, 25 patients suffered from idiopathic aqueductal stenosis (35.7%), 22 from PHH (31.4%), 8 from tumor-related hydrocephalus (11.4%), 5 from fourth ventricle outlet obstruction (7.1%), 6 from Chiari malformation-type II with myelomeningocele (8.6%), and 4 from post-infection hydrocephalus (5.7%). Fifteen patients had a VP shunt prior to ETV. Mean follow-up period was 68.9 months (range 3–270 months). The ETV procedure was successful in 29 out of 70 patients (41.4%). The 3 patients who received a Re-do for ETV failed during follow-up and received a VP shunt.

### Postoperative complications

In our study, we encountered 16 patients out of 70 who developed a complication after the ETV procedure or during follow-up (22.9%). We found 2 patients who had an intraoperative bleeding (2.9%): one was during ventriculostomy and the other bled from a pineal region tumor after ETV and biopsy; we found in 5 patients a CSF collection (7.1%), in 2 of them they suffered from external CSF leak; 4 patients suffered from CSF infection in the form of meningitis (5.7%) in 2 patients and ventriculitis in another 2 patients. Two patients suffered from VP shunt infection (2.9%) which was placed after failed ETV, one patient suffered from subdural hygroma (1.4%) which was conservatively managed. Two patients suffered temporarily from diabetes insipidus after ETV procedure (2.9%). All the complications observed were in the patients who showed finally a failure of ETV. For details, see Table 1.

**Table 1:** Complications after ETV

Complications	No. of patients (percentage)
Bleeding	2 (2.9%)
CSF collection	3 (4.3%)
CSF fistula	2 (2.9%)
Meningitis	2 (2.9%)
Ventriculitis	2 (2.9%)
VP shunt infection	2 (2.9%)
Hygroma	1 (1.4%)
Diabetes insipidus	2 (2.9%)

### ETV success tailored to the etiology of hydrocephalus and the patient age

**Fig. 1:**
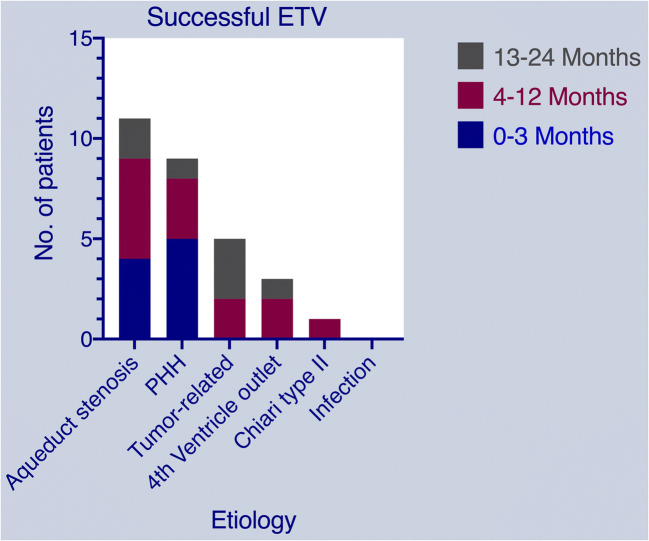
Stacked column chart showing outcome of ETV according to etiology

ETV was successful in 11/25 patients (44%) with idiopathic aqueductal stenosis, 9/22 patients (40.9%) with PHH, 5/8 patients (62.5%) with tumor-related hydrocephalus, 3/5 patients (60%) with fourth ventricle outlet obstruction, 1/6 patients (16.7%) with Chiari-type II, and none (0/4) with post-infection hydrocephalus (see Fig. 1). None of the Re-do ETV was successful. There were no statistically significant differences between the groups (*p* = 0.75).

**Fig. 2:**
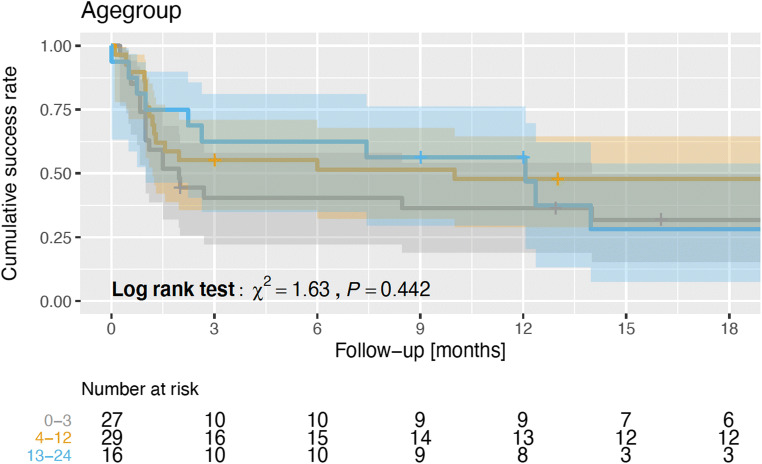
ETV failure over the follow-up time indicated as Kaplan-Meier estimators using the different age groups. Log-rank tests were conducted to check the hypothesis of the equality of event time distributions

According to age groups, the first age group (below 3 months age) showed success rate 33.3% (9 out of 27 patients: 4 with aqueduct stenosis and 5 with PHH), second age group (4–12 months age) showed 46.4% success rate (13 out of 28 patients: 5 with aqueductal stenosis, 3 with PHH, 2 with tumor-related hydrocephalus, 2 with fourth ventricle outlet obstruction, and 1 with Chiari malformation-type II), and finally the third age group (13–24 months age) showed 46.7% success rate (7 out of 15 patients: 2 with aqueductal stenosis, 1 with PHH, 3 with tumor-related hydrocephalus, 1 with fourth ventricle outlet obstruction, see Fig. 2). There were no statistically significant differences between the groups (*p* = 0.88).

### Risk-factor analysis for the ETV success

**Fig. 3:**
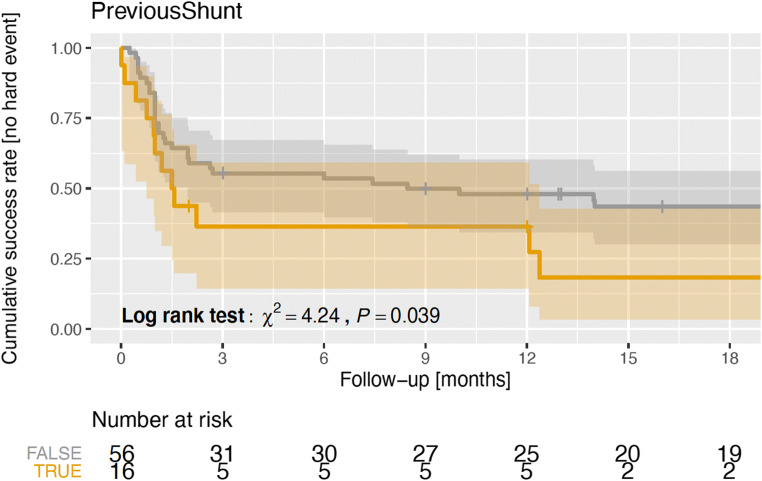
ETV failure over the follow-up time indicated as Kaplan-Meier estimators using the differentiation between patients with a previous shunt or not are shown. Log-rank tests were conducted to check the hypothesis of the equality of event time distributions

In the univariable time-to-event analysis, we found a statistically significant higher risk for an ETV failure in patients with a previous shunt (log-rank-test, *p* < 0.05) (Fig. 3). The time-to-event analysis did not show any effect on the ETV success by the patient age, the diagnosis, or the participating study center.

The univariable logistic regression could not confirm a previous shunt as a predictor for the failure 6 months after ETV (*p* = 0.21). Also, it did not show any other significant predictive factors. The multivariable logistic regression adjusted for patient age, gender, the participating study center, as well as a VP shunt in the patient history did not show a significant predictor for an ETV failure. However, the age group III (patients from 13 to 24 months) failed to have a statistically significant better outcome (*p* = 0.053). Age included as a continuous and as a categorical variable made no distinction in the results.

## Discussion

### Summary of the main results

The present study reveals an overall success rate of ETV in infants of 41.4%. The difference of success was not statistically significant, but clinically relevant separating more favorable results in patients suffering tumor-related hydrocephalus and fourth ventricular outlet obstruction from poorer results in patients suffering postinfectious hydrocephalus or hydrocephalus associated with myelomeningocele. Patients aged 4 months or older at time of procedure had an improved outcome. A VP shunt implantation prior to ETV seems to be a risk factor for ETV failure in infants.

### Limitations of the study

One of the big limitations of the study, being a retrospective one from 4 university centers, is the diversity of the operators and not considering the factor of surgical experience. Although the definition of failure of ETV is not easy to establish, we agreed on considering failure whenever a VP shunt is applied in further follow-up. There was no standardized surgical protocol. Therefore, there was significant subjectivity in the decision to place a VP shunt or do a Re-ETV in certain patients. This decision was left to the judgment of the surgeon.

### ETV success in children below 2 years of age

Baldauf et al. [1] reported on a series of 21 patients with age ranging from 9 days to 15 months treated with ETV. It was successful in 9 patients: four patients with idiopathic aqueductal stenosis, in two with other congenital anomalies, in one PHH, and in two with a tumor-related hydrocephalus. They concluded that the success of ETV in children younger than 2 years of age is dependent on both age and etiology. They reported an overall success rate of 43%. In 37.5% of the children younger than 1 year of age, ETV was successful. ETV in patients with hydrocephalus due to idiopathic aqueductal stenosis seems to be more beneficial than in other causes of hydrocephalus as shown in our study.

Kulkarni et al. [21] calculated the success rate of ETV according to the endoscopic third ventriculostomy success score (ETVSS) which considered the factor of age and etiology as well as previous VP shunt application. Across all ETVSS strata, the risk of ETV failure becomes progressively lower compared with the risk of shunt failure with increasing time from the surgery. Since then, there have been many studies carried out on assessing the validity of ETVSS in pediatric population. Labidi et al. [22] tried to assess the validity in a mixed group of 168 adult and pediatric patients. All the patients were older than 2 years of age. They reported that the ETVSS did not show adequate discrimination but demonstrated excellent calibration in this population of patients 2 years and older. Chowdhury et al. [23] assessed the success rate of ETV prospectively in infant age group. Fourteen out of 17 patients (82.35%) showed overall clinical improvement. In this series, an average ETV success score (ETVSS) was 52.35 (range: 40–70) and overall success rate was 82.35%. They concluded that ETVSS did not correlate with the outcome of ETV in infants with aqueductal stenosis. To our knowledge, there has been until now no studies assessing ETVSS with variant etiologies in children during the first 2 years of life. This is possibly caused by the expected low success rate below 6 months of age considering ETVSS.

Klebe et al. [24] tried to reconcile our knowledge of germinal matrix hemorrhage (GMH) with or without intraventricular hemorrhage (IVH) and the consequent PHH development with the current hydrodynamic theory of hydrocephalus. PHH after GMH may be obstructive, non-communicating hydrocephalus during the acute phase due to the hematoma, but generally develops as chronic communicating hydrocephalus into adolescence and adulthood. Indeed, many GMH/IVH studies suggest PHH is a consequence of obstructions within the ventricular system and subarachnoid drainage pathways due to thrombi, gliosis, and fibrosis. These are not merely obstructing CSF passages but are altering barrier dynamics in the microvasculature and ependymal lining, altering CSF dynamics, and lead to PHH development. This goes with our results as patients with PHH represented the least favorable etiological group with success rate of 40.9% and only better than patients with Chiari malformation-type II or post-infection which showed success rate of 16.7% and 0% respectively. This could be explained due to the alterations in the microvasculature previously mentioned.

Duru et al. [20] reported their experience with ETV in 51 children below 16 years of age; they reported an overall success rate of 80% (40/51) for all etiologies and ages. In patients < 6 months of age, the success rate was 56.2% (9/16), while 6–24 months of age was 88.9% (16/18) and > 24 months was 94.1% (16/17). The highest success rate was obtained in aqueductal stenosis. Success rate of PHH, postinfectious, and spina bifida-related hydrocephalus was 60% (3/5), 50% (1/2), and 14.3% (1/7), respectively. In comparison to our results, all our patients were below 24 months of age at time of ETV. We found the overall success rate was 41.4% with a relatively higher success rate in the 2 age groups above 3 months of age (4–12 months and 13–24 months with 46.4% and 46.6% respectively). We reported also better results in cases of tumor-related hydrocephalus (62.5%), tetraventricular hydrocephalus (60%), aqueductal stenosis (44%), and PHH (40.9%). The success rate in association with meningomyelocele was quite lower (16.7%) and only failure after infection.

### Pathophysiological considerations of the CSF system in infants

Understanding the CSF physiology is still evolving and incomplete. In the traditional bulk flow model described by Dandy over a century ago, CSF is secreted by the choroid plexus epithelium in the ventricles, flows into the subarachnoid spaces, and enters the cerebral venous system via the arachnoid granulations. In this model, obstruction of CSF flow within the ventricles is classified as obstructive or non-communicating hydrocephalus, whereas obstruction of CSF flow or its absorption in the subarachnoid spaces is known as communicating hydrocephalus [25, 26].

Oi and Di Rocco [27] proposed a new classification for hydrocephalus with special reference to the CSF circulation in the minor CSF pathway, i.e., “minor pathway hydrocephalus.” In large mammals and humans, the arachnoid granulation (Pacchionian body) appears in postnatal life or just before birth microscopically as villi and begins to function as the CSF reabsorption route in the later infantile age [27]. Due to the missed functional development of the arachnoid granulation, the CSF dynamics may be maintained at the minor pathway with the drainage route via perineural space to the lymphatic system [27, 28]. This “minor CSF pathway” is the main route for the CSF dynamics both in rodents or small mammals and developing immature brain in humans. Iliff et al. [29] further characterized this pathway in rodents using in vivo two-photon imaging and coined the term “glymphatic system.”

Studies on the meningeal lymphatic vessels development in mice using enhanced green fluorescent protein (GFP) in lymphatic endothelium showed that the first meningeal lymphatic vessels were observed just before birth at the base of the skull around the foramen magnum. After birth, lymphatic vessels tend to extend along the middle meningeal artery and continue further growth. At 1 month of age, the meningeal lymphatic vessels are developed in all parts of the skull. Comparison of the reporter mice at 1 month and 2 years of age revealed their similar patterning around blood vessels, cranial, and spinal nerves [30, 31]. In a similar way, the glymphatic system may also in humans be in the phase of development after birth. Taking into consideration its role in CSF circulation and absorption during the early infancy, when the Pacchionian bodies, i.e., arachnoid villi are still not well-developed and not functioning, we expected that the outcome of ETV in early infancy, i.e., the first 3 months of life would be relatively lower than in later infancy due to the immaturity of both the glymphatic system as well as the arachnoid villi. This goes with our results regarding success rate in the age group less than 3 months which showed a lower success rate (33%) in comparison to later infancy with success rate of almost 46.5% (20/43) regardless the etiology. However, this difference did not reach statistical significance. As the multivariate analysis did show, the age group 13–24 months had the best outcome and missed statistical significance very brief.

## Conclusion

The present study reveals an overall success rate of ETV in infants of 41.4%. The difference of success was not statistically significant, but clinically relevant separating more favorable results in patients suffering tumor-related hydrocephalus and fourth ventricular outlet obstruction from poorer results in patients suffering postinfectious hydrocephalus or hydrocephalus associated with myelomeningocele. Patients aged 4 months or more had an improved outcome. A VP shunt implantation prior to ETV seems to be a risk factor for ETV failure in infants.
